# Inflammation-related pathways involved in damaged articular cartilage of rats exposed to T-2 toxin based on RNA-sequencing analysis

**DOI:** 10.3389/fgene.2022.1079739

**Published:** 2022-12-05

**Authors:** Longyan Shi, Qiuliang Liu, Heying Yang, Qi Wang, Jiaxiang Wang, Yingzhong Fan

**Affiliations:** Department of Pediatric Surgery, First Affiliated Hospital of Zhengzhou University, Zhengzhou, Henan, China

**Keywords:** T-2 toxin, articular cartilage, inflammatory response, RNA-seq, IL-17 signaling pathway, TNF signaling pathway

## Abstract

Many studies have shown that ingestion of the T-2 toxin is harmful to articular cartilage. However, the mechanisms underlying damaged articular cartilage induced by T-2 toxin have not been elucidated. Twenty-four SD rats were randomly divided into T-2 toxin and control groups. In the control group, the 12 rats were administered 4% absolute ethanol by gavage, and in the T-2 toxin group, the 12 rats were administered T-2 toxin (100 ng/g, BW/day) by gavage. After the rats were sacrificed, the knee joints were collected, and RNA was extracted using TRIzol reagent for RNA sequencing (RNA-seq). Differentially expressed mRNA was identified based on *p* < 0.05 and | log_2_ (fold change) | > 1. The T-2 toxin-related genes were obtained from the GeneCards database. An online tool (https://www.bioinformatics.com.cn) was used for enrichment analysis. Hematoxylin and eosin (H&E) staining was used to observe damaged articular cartilage, and immunohistochemical (IHC) staining was used to validate differentially expressed proteins. The H&E staining shows the number of cells decreased significantly, and the arrangement of chondrocytes became disordered in the T-2 toxin group. RNA-seq analysis identified 195 upregulated and 89 downregulated mRNAs in the T-2 toxin group. The top immune-related biological processes (Gene Ontology) were regulation of hormone secretion, regulation of peptide hormone secretion, and regulation of transcription involved in cell fate commitment. KEGG pathway enrichment analysis revealed that the IL-17 and tumor necrosis factor signaling pathways were significantly expressed, and the IL-17 signaling pathway was also identified in the enrichment analysis of T-2 toxin-related genes. Also, Mmp3, Tnf, Mapk10, Ccl11, Creb5, Cxcl2, and Cebpb were significantly enriched in the two pathways. The immunohistochemical staining showed that the levels of Mmp3 and Tnf proteins were significantly increased in the T-2 toxin group, which was consistent with the RNA-seq results. This study revealed the critical roles of IL-17 and TNF signaling pathways in damaged cartilage induced by T-2 toxin.

## Introduction

The T-2 toxin is the most toxic class A trichothecene mycotoxin produced by *Fusarium*. It is widely distributed in nature and mainly contaminates cereals and grains, such as wheat, barley, oats, rice, maize, and sorghum, and is harmful to humans and poultry. At present, it is believed that the T-2 toxin can cause a variety of human and animal diseases such as alimentary toxic aleukia and Kashin–Beck disease (KBD) (Lutsky et al., 1978; Li et al., 2016). The Soviet Union scholar proposed the hypothesis of food mycotoxin contamination in 1964, and the causative organism was considered to be a toxigenic strain of *Fusarium sporotrichiella. var. poae Bilai* (Sokoloff, 1988). The rats fed with *Fusarium* showed epiphyseal lesions and femur shortening, while the rats in the control group developed normally (Guo et al., 2014). In 1990s, Yang Jianbo proposed a relationship between the T-2 toxin and KBD according to epidemiological investigations (Dianjun Sun; Wang et al., 2020). In 1995, he investigated edible grains of severe KBD areas in Shaanxi and Sichuan provinces and the commercially available grains in non-KBD areas from Henan, Hubei, Hunan, and Guangxi provinces. The results showed the concentration of T-2 toxin (ranging from 2.0 to 1,549.4 ng/g) in 10 out of 15 family-grown flour samples in the KBD areas was positive, which was significantly higher than that of the commercial flour samples in non-KBD areas (ranging from 2.0 to 885.3 ng/g). Meanwhile, the study also confirmed the threshold concentration of T-2 toxin in flour should not exceed 300 ng/g (Yang et al., 1995). Therefore, the concentration of T-2 toxin in the flour and corn meal of families affected by KBD resulted in an abnormal aggregation phenomenon.

According to the food mycotoxin contamination hypothesis, the grain harvest season is rainy, and thus, fungi contaminate the grain during harvesting, drying, and storage. As healthy children in the KBD areas consumed fungus-contaminated grains, the T-2 toxin selectively damaged the developing articular cartilage, resulting in damage to the membrane system of chondrocytes, inhibition of DNA synthesis, and induction of chondrocytes apoptosis (Guo et al., 2014). When chondrocytes were incubated with T-2 toxin, the growth rate decreased, the protein and DNA contents were reduced, levels of collagen II and proteoglycan decreased, expression of matrix metalloproteinase 13 increased, and chondrocyte apoptosis was induced (Liu et al., 2021; Li H. N. et al., 2022). When the chicks were fed diets containing T-2 toxin for 5 weeks, their articular cartilage underwent degenerative changes, which caused deep necrosis of the epiphyseal plate cartilage (Yu et al., 2022). Wistar rats were treated with T-2 toxin (100 ng/kg) for 10 months, and the results showed chondrocyte degeneration/necrosis and loss, chondrocyte clones, and loss of proteoglycan staining of articular cartilage in the femorotibial cartilage. The rats exposed to T-2 toxin showed degenerative lesions in articular cartilage similar to spontaneous osteoarthritis (Wang et al., 2011; Yu et al., 2022). However, the deep necrosis of articular cartilage in human KBD has not been replicated.

A previous study examined the DNA methylation dynamics of human chondrocytes treated with T-2 toxin and found that the dysfunction of the MAPK pathway was involved in the damaged human chondrocyte (Yang et al., 2022). However, previous studies have only been performed at the cellular level and have not been validated in cartilage tissues. In this study, SD rats were treated with T-2 toxin (100 ng/g) for 4 weeks, and the articular cartilage tissues were collected. RNA transcription sequencing technology was used to explore the mechanism of damaged articular cartilage induced by the T-2 toxin.

## Methods and materials

### Establishment and treatment of animal model

A total of 24 SPF-grade SD rats (aged 4 weeks, 60−80 g) were purchased from the Henan Provincial Laboratory Animal Center. They were maintained in a spacious animal house and fed a standard mouse diet and water *ad libitum*. The feed and ultrapure water were kept for 1 week, and after weighing, the rats were randomly divided into the control and T-2 toxin groups. The 12 rats were administered 4% absolute ethanol by gavage in the control group, and the 12 rats were administered T-2 toxin (100 ng/g; BW/day; J&K Scientific LLC, USA) by gavage in the T-2 toxin group (Yu et al., 2022). During this period, the weight and other indicators of their growth and development were recorded. After treatment for 1 month, three rats in each group were anesthetized with urethane, and three articular cartilages were used to extract RNA. All animal studies were reviewed and approved by the Animal Ethics Research Committee of Zhengzhou University (ZZUIRB 2021-69).

### Hematoxylin–eosin (H&E) staining

After the rats were sacrificed, the knee joints were collected, fixed in 4% paraformaldehyde for 24 h, and decalcified with 10% EDTA for 1 month. The decalcified knee joints were embedded in paraffin and sectioned. The tissue sections were stained with H&E. Finally, the stained tissue sections were dehydrated and sealed with neutral gum, and the collected images were observed under a microscope.

### Total RNA extraction

RNA was extracted using the TRIzol reagent (Thermo Fisher Scientific, USA) for RNA sequencing (RNA-seq). After collecting the RNA samples, the concentration was accurately quantified using Qubit, and the ratios of OD_260/280_ and OD_260/230_ were detected using a Nanodrop 2000 spectrophotometer (Thermo Fisher Scientific, USA). Then, the degree of RNA degradation was detected by agarose gel electrophoresis and an Agilent 2100 Bioanalyzer (Agilent Technologies, USA).

### Construction of the DNA-specific library

An appropriate amount of total RNA was used to remove rRNA, and the RNA was purified and fragmented. Reverse transcription was used to synthesize the first strand cDNA and second strand cDNA. Next, a single adenylate “A” was added to the 3′ ends on both sides of the modified double-stranded cDNA to prevent self-ligation of the flat ends between the cDNA fragments. Ligation buffer and a double-stranded sequencing linker were added to the aforementioned reaction system, and the Illumina sequencing linker was connected to both ends of the DNA library using T4 DNA ligase (NEB, UK). The Agencourt SPRI select Nucleic Acid Fragment Screening kit was used to screen the fragment size while the library was being purified, and a two-step screening method was used to select the original library with a fragment peak of 300 bp. In a 50-μl reaction system, high fidelity polymerase was used to amplify the original library to ensure a sufficient total number of libraries. The constructed library was qualified using Qubit and an Agilent 2100 Bioanalyzer (Agilent Technologies, USA) and then sequenced using an Illumina HiSeq 2500 System (Illumina, USA).

### Differential expression mRNA analysis

Deseq2 software was used to analyze differentially expressed mRNA in the T-2 toxin and control groups. *p*-values <0.05 and | log_2_ (fold change) | > 1 were considered differentially expressed mRNAs, log_2_ (fold change) > 1 was labeled as an upregulated mRNA, and log_2_ (fold change) < −1 was labeled as a downregulated mRNA.

### Enrichment analysis

Gene Ontology (GO) is an international standard classification system for gene functions. Through GO enrichment analysis, significantly differentially expressed genes were classified according to cellular components, molecular functions, and biological processes. KEGG (Kyoto Encyclopedia of Genes and Genomes) is a vital public database of classical pathways for experimental verification. Pathway enrichment analysis takes the KEGG pathway as the unit and applies a hypergeometric test to determine which pathways are significantly enriched in a specific gene set compared to the whole genome background. The analysis was based on differentially expressed genes marked as up and down, and an online tool (https://www.bioinformatics.com.cn) was used to perform GO and KEGG enrichment analyses (Luo & Brouwer, 2013).

### T-2 toxin-related genes from GeneCards

The T-2 toxin-related genes were obtained from the GeneCards (https://www.genecards.org/) database. The relevance scores of >5 were used as the screening criteria (Safran et al., 2022). KEGG pathways were plotted using a free online platform for data analysis and visualization (https://www.bioinformatics.com.cn).

### Immunohistochemical (IHC) staining

The dewaxed and rehydrated tissue sections were placed in a repair box filled with citric acid (pH 6.0) antigen repair buffer. Next, 3% hydrogen peroxide was used to inhibit the endogenous peroxidase activity. After blocking with 3% bovine serum albumin for 30 min at room temperature, the slices were incubated with primary anti-Mmp and anti-Tnf antibodies (1:500 dilution, Wuhan Servicebio Biotechnology) overnight at 4°C and then incubated with horseradish peroxidase–conjugated secondary antibodies for 50 min at room temperature. Next, the slices were washed with phosphate buffered saline, developed, and sealed after re-staining the nuclei with hematoxylin. Semi-quantitative analysis of the acquired images was performed using Image Pro Plus 6.0.

## Results

### Influence of cartilage tissues treated with T-2 toxin

**FIGURE 1:**
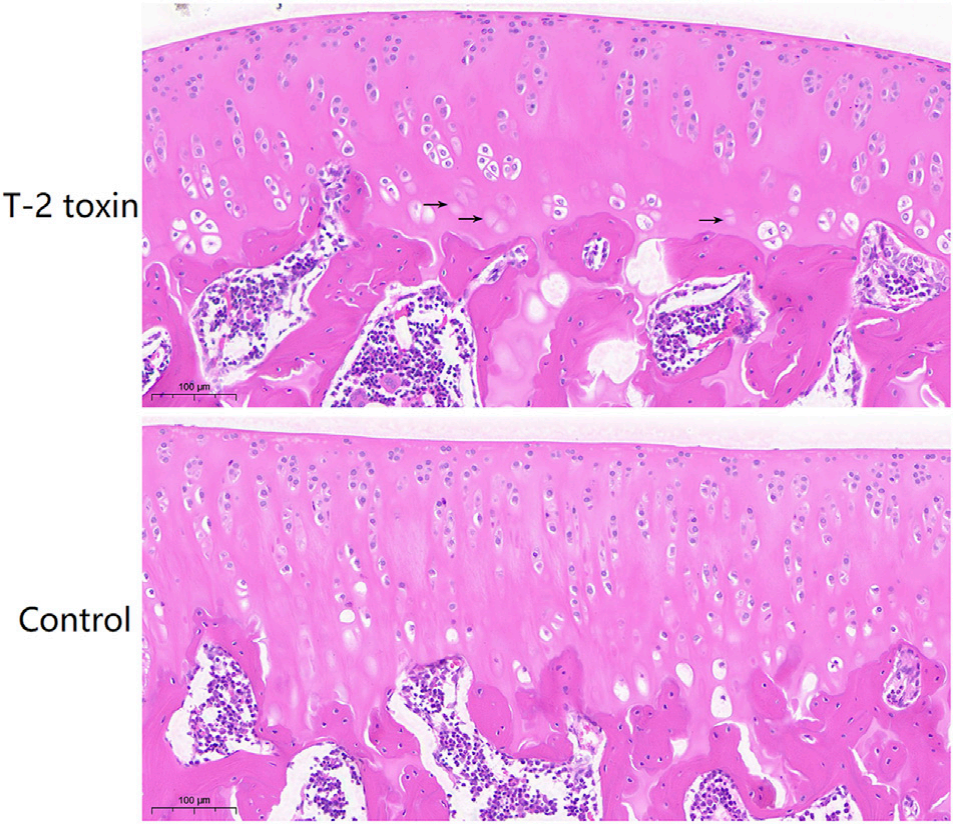
Pathological changes of rat cartilage tissues in the T-2 toxin and control groups using H&E staining. The black arrows indicated chondrocytes without the nucleus.

The H&E staining results show the histopathologic changes of rat cartilage tissues in each group (Figure 1). In the control group, chondrocytes were abundant and arranged in an orderly manner from top to bottom, and the morphology of the cells and nuclei was normal. Abnormalities were not observed in the control group. In the T-2 toxin group, the number of cells decreased significantly, and the arrangement of chondrocytes became disordered. The hypertrophic chondrocytes were clustered, and the physalide phenomenon caused by the loss of the nucleus also appeared.

### Identification of differentially expressed mRNA

**FIGURE 2:**
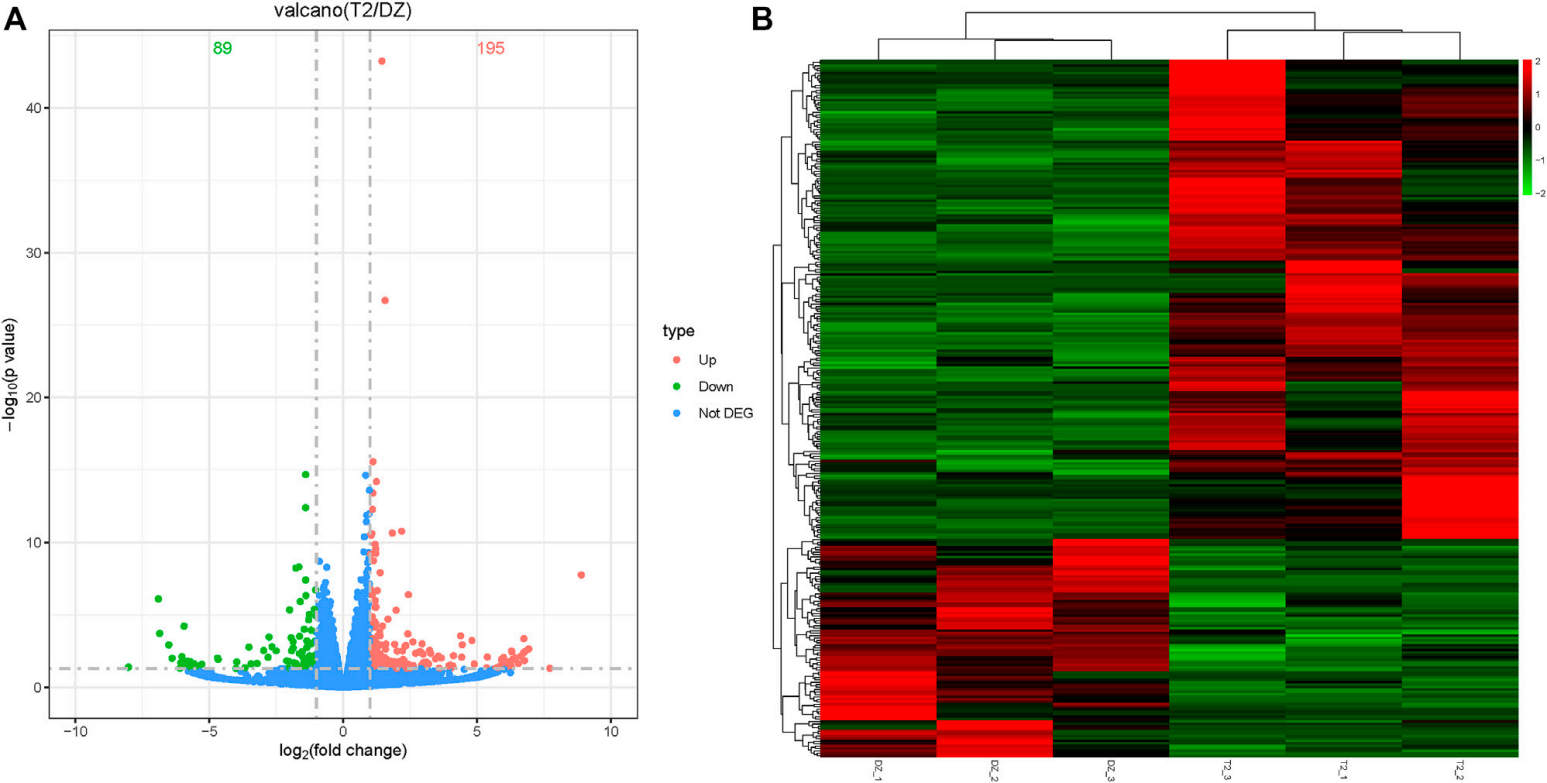
Differentially expressed mRNA analysis and cluster analysis. **(A)** Volcano of differentially expressed mRNA in the T-2 toxin group compared with the control group. Green represents downregulated mRNA, and orange represents upregulated mRNA. **(B)** Hierarchical clustering of differentially expressed mRNA in the T-2 toxin group compared with the control group. Green represents downregulated mRNA, and red represents upregulated mRNA.

Analyses of differentially expressed mRNAs showed that 284 significant differentially expressed mRNAs were identified in the T-2 group compared to the control group. Of these, 195 mRNAs were significantly upregulated, and 89 mRNAs were downregulated (Figure 2A). Cluster analysis was performed to identify differentially expressed mRNAs between the T-2 and control groups. In Figure 2B, each column represents a different sample, and each row represents the expression levels of mRNA in different colors: up (red) or down (green).

### GO analysis

**FIGURE 3:**
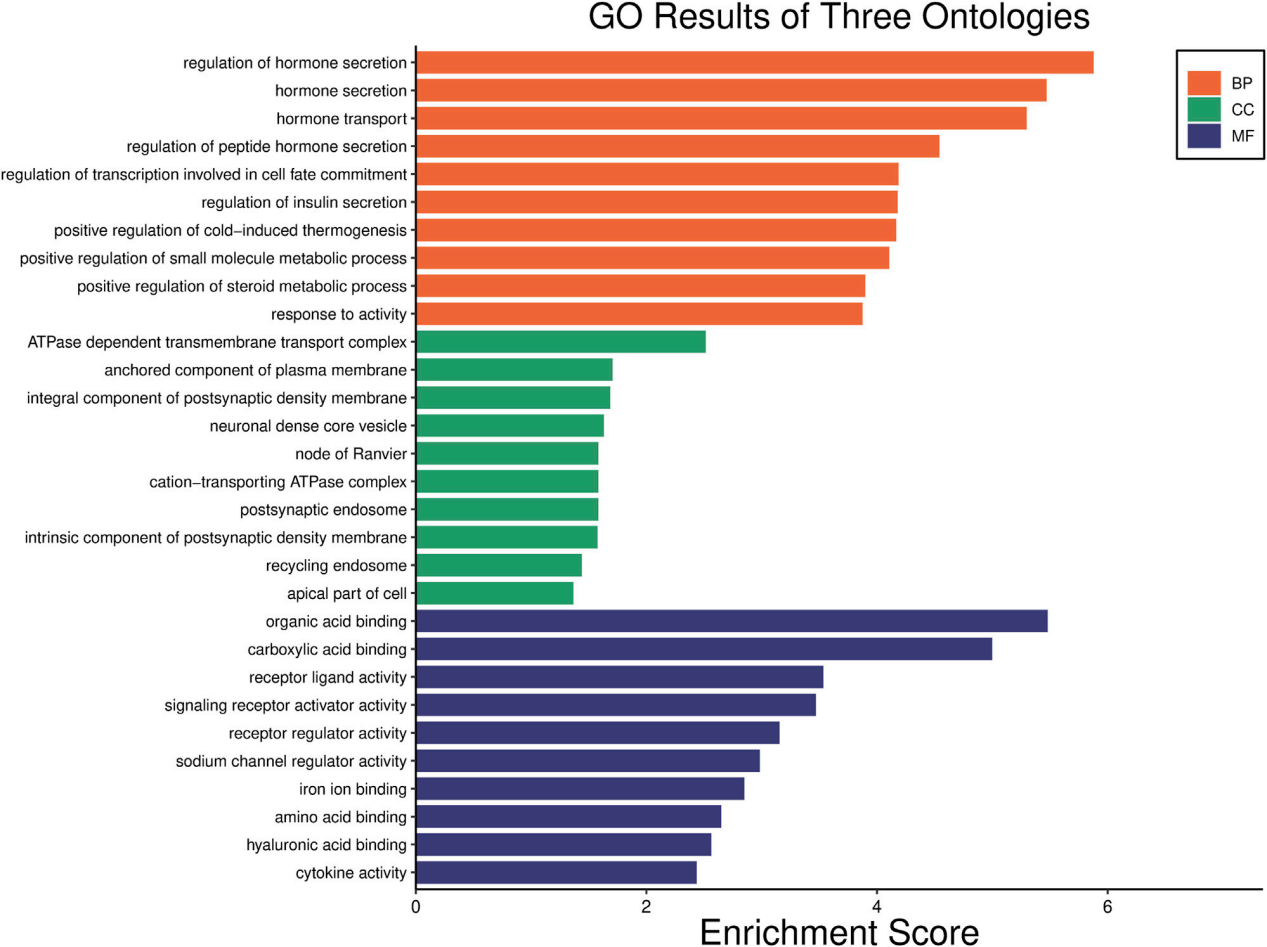
Gene Ontology (GO) functional classification. The significant GO terms of differentially expressed genes in the T-2 toxin group compared with the control group. The orange, green, and blue bars represent the biological process, cellular component, and molecular function, respectively.

The GO results were divided into three categories (namely, biological process, cellular component, and molecular function). As shown in Figure 3, the top immune-related biological processes were the regulation of hormone secretion, regulation of peptide hormone secretion, regulation of transcription involved in cell fate commitment, positive regulation of the small molecule metabolic process, and response to activity. The top immune-related cellular components were the ATPase-dependent transmembrane transport complex, an anchored component of the plasma membrane, the cation-transporting ATPase complex, the recycling endosome, and the apical part of the cell. The top immune-related molecular functions were receptor–ligand activity, signaling receptor activator activity, receptor regulator activity, sodium channel regulator activity, iron ion binding, and cytokine activity.

### KEGG pathway analysis

**FIGURE 4:**
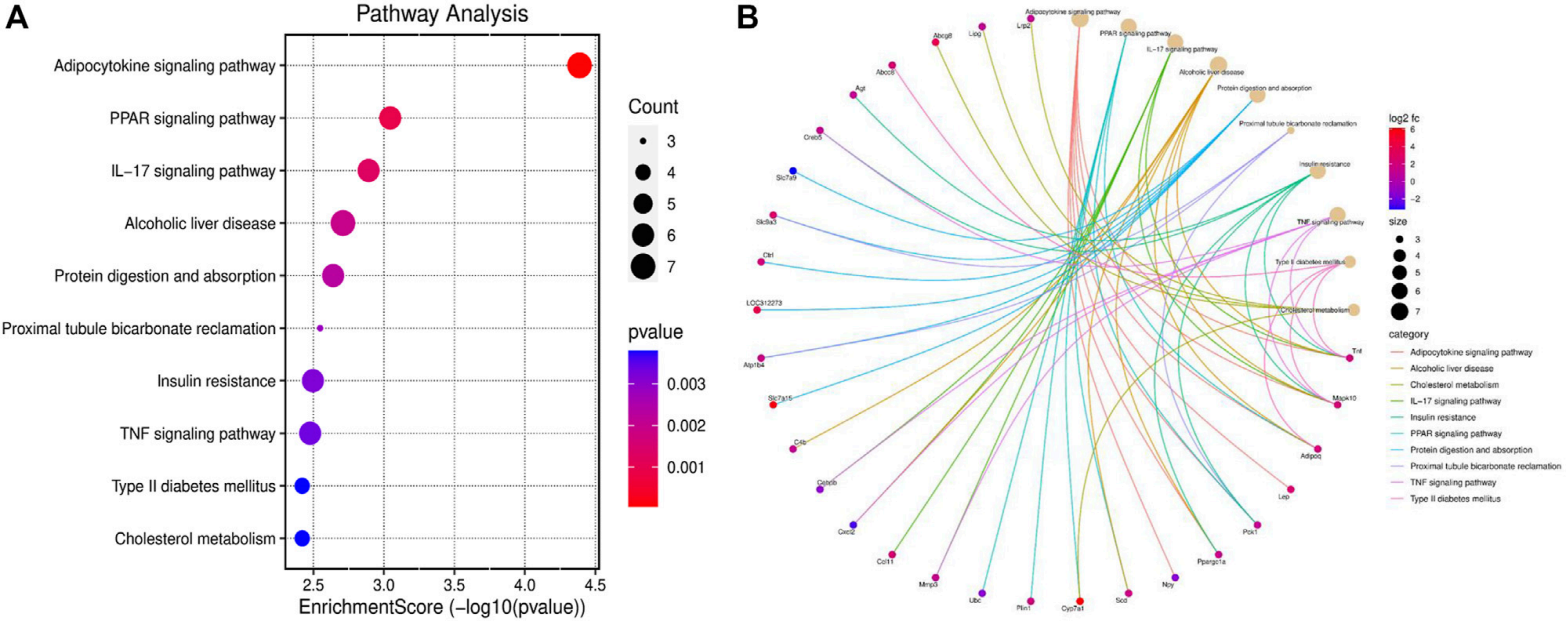
KEGG pathway enrichment analysis (top 10). **(A)** Top 10 significant KEGG pathways of differentially expressed genes in the T-2 toxin group compared with the control group. **(B)** Relationship between significant pathways and enriched genes, and different colors represent different pathways. *p*-value <0.05 was considered significant pathways.

**FIGURE 5:**
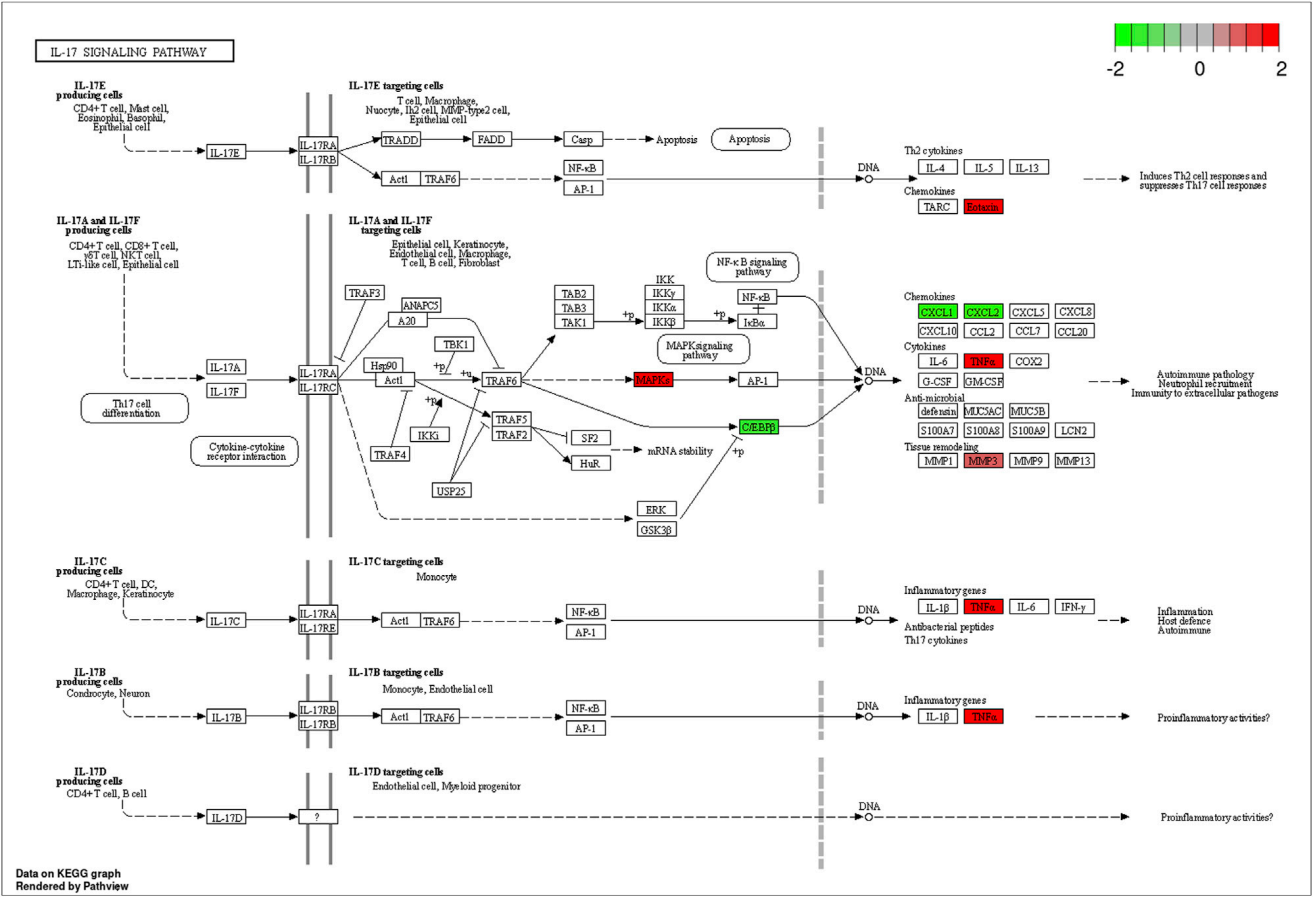
Differentially expressed genes enriched in the path diagram of the IL-17 signaling pathway.

**FIGURE 6:**
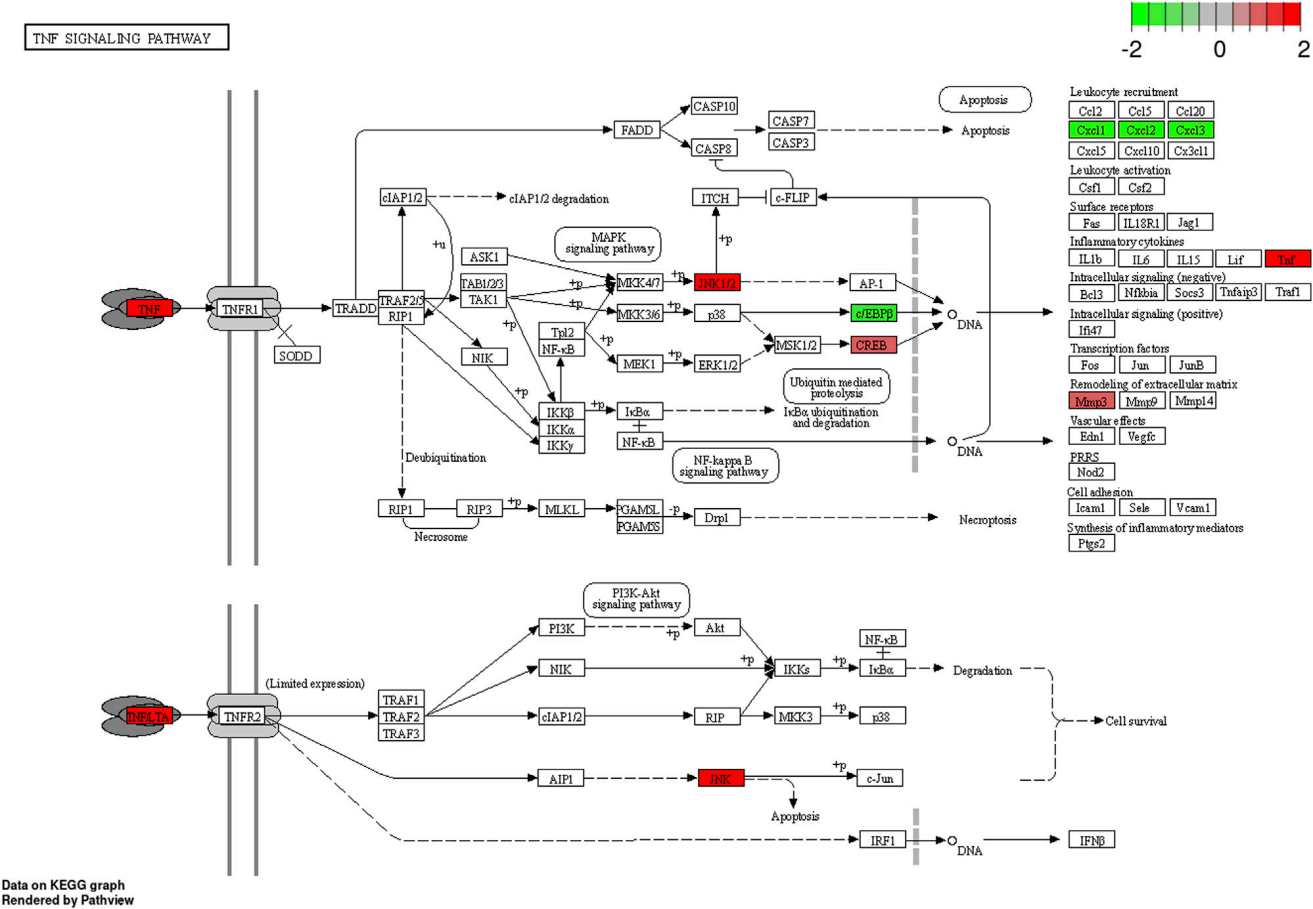
Differentially expressed genes enriched in the path diagram of the TNF signaling pathway.

The KEGG pathway enrichment analysis identified significant pathways. According to the enrichment score, the top 10 significant pathways were the adipocytokine signaling pathway, the PPAR signaling pathway, the IL-17 signaling pathway, alcoholic liver disease, protein digestion and absorption, proximal tubule bicarbonate reclamation, insulin resistance, the TNF signaling pathway, type II diabetes mellitus, and cholesterol metabolism (Figure 4A). Figure 4B shows the relationship between significant pathways and enriched genes, and different colors represent different pathways. The path diagrams of the IL-17 signaling pathway (Figure 5) and the TNF signaling pathway (Figure 6) were significantly correlated with the damaged articular cartilage exposed to T-2 toxin. Seven identified genes were enriched in the IL-17 and TNF signaling pathways (Table 1).

**TABLE 1:** Differentially expressed genes enriched in the IL-17 and TNF and signaling pathways.

Gene symbol	Gene name	log_2_ fold change	*p*-value	Type
Mmp3	Matrix metallopeptidase 3	1.10	5.38 × 10^−5^	Up
Tnf	Tumor necrosis factor	2.03	2.08 × 10^−2^	Up
Mapk10	Mitogen-activated protein kinase 10	2.23	1.21 × 10^−2^	Up
Ccl11	C-C motif chemokine ligand 11	2.26	1.34 × 10^−2^	Up
Creb5	cAMP-responsive element-binding protein 5	1.09	5.28 × 10^−13^	Up
Cxcl2	C-X-C motif chemokine ligand 2	−2.77	3.34 × 10^−4^	Down
Cebpb	CCAAT/enhancer-binding protein-beta	−1.26	6.55 × 10^−3^	Down

### T-2 toxin-related gene enrichment analysis

**FIGURE 7:**
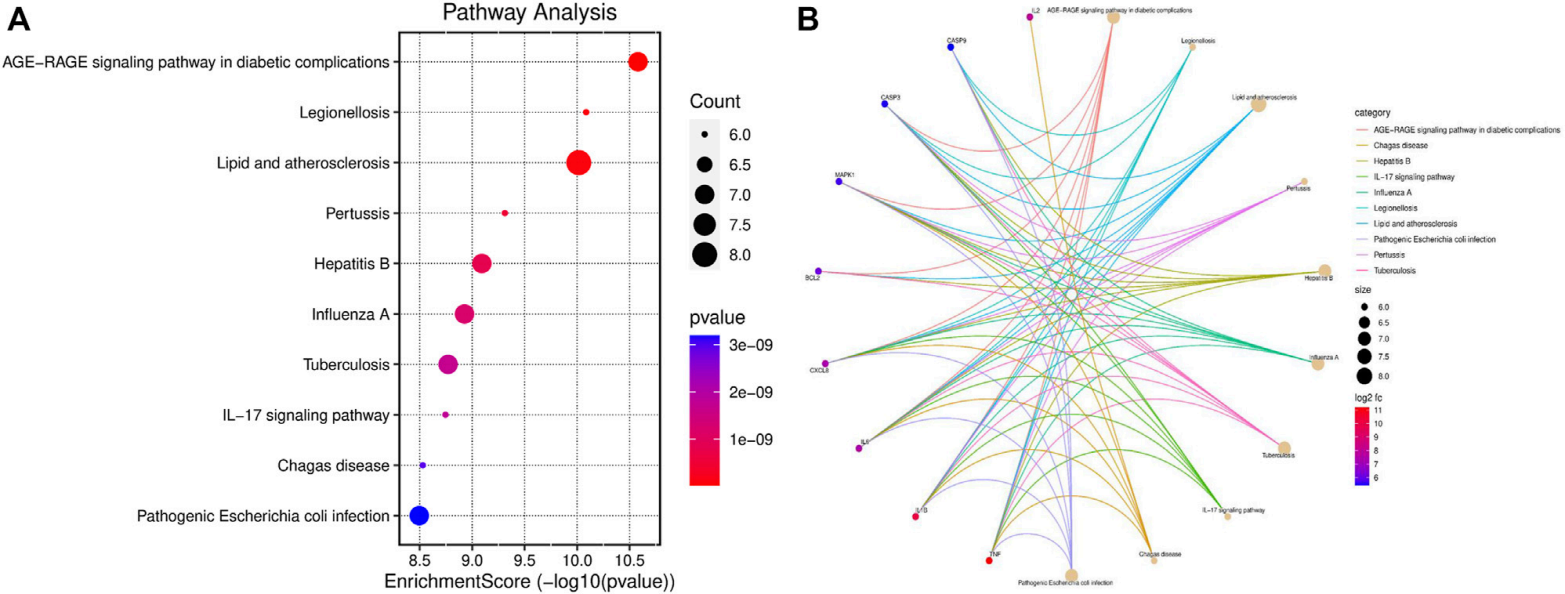
KEGG pathway enrichment analysis (top 10). **(A)** Top 10 significant KEGG pathways of T-2 toxin-related genes. **(B)** Relationship between significant pathways and enriched genes, and different colors represent different pathways. *p*-value <0.05 was considered significant pathways.

In the GeneCards database, the 13 identified T-2 toxin-related genes (Table 2) were screened with a score greater than 5. KEGG pathways were enriched using an online tool (https://www.bioinformatics.com.cn). The IL-17 signaling pathway was significantly correlated with T-2 toxin (Figure 7A). The enriched genes (TNF, IL1B, IL2, IL6, and CXCL8) are presented in Figure 7B.

**TABLE 2:** T-2 toxin-related genes from GeneCards based on the score more than 5.

Gene symbol	Gene name	GIFtS	GC ID	Score
TNF	Tumor necrosis factor	54	GC06P087731	11.21
IL1B	Interleukin 1 beta	50	GC02M112829	10.09
ALB	Albumin	52	GC04P073397	8.36
IL2	Interleukin 2	51	GC04M122451	7.99
FURIN	Furin, paired basic amino acid cleaving enzyme	50	GC15P090868	7.68
IL6	Interleukin 6	53	GC07P022725	7.56
CXCL8	C-X-C motif chemokine ligand 8	44	GC04P073740	7.29
PARP1	Poly (ADP-ribose) polymerase 1	53	GC01M226360	6.45
BCL2	BCL2 apoptosis regulator	53	GC18M063123	6.15
EGF	Epidermal growth factor	54	GC04P109912	5.82
MAPK1	Mitogen-activated protein kinase 1	54	GC22M021759	5.67
CASP3	Caspase 3	52	GC04M184627	5.48
CASP9	Caspase 9	49	GC01M015491	5.43

Abbreviation: GIFtS, GeneCards Inferred Functionality Score; GC ID, GeneCards identifiers.

### IHC staining

**FIGURE 8:**
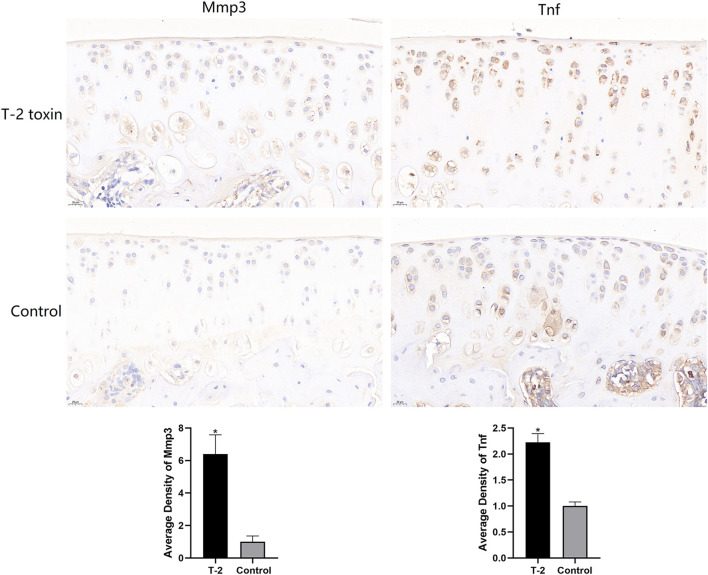
Immunohistochemistry staining of knee joint cartilage of rats in the T-2 toxin and control groups. All data represent mean ± SD, and *p < 0.05 was considered significant.

The expression levels of Mmp3 and Tnf proteins were determined by IHC staining. As shown in Figure 8, the positive expression of Mmp3 and Tnf proteins in the T-2 toxin group was significantly higher than that in the control group, and the expression levels of Mmp3 and Tnf proteins were increased in the T-2 toxin group (*p* < 0.05).

## Discussion

It is well known that T-2 toxin is harmful to human health. T-2 toxins can cause cytotoxicity, genotoxicity, immunotoxicity, hepatotoxicity, gastrointestinal toxicity, skeletal toxicity, nephrotoxicity, reproductive toxicity, and neurotoxicity. It damages the immune organs, such as the liver, digestive tract, bones, and kidneys, leading to pathological changes and impaired physiological functions of these organs (Li S. J. et al., 2022). Furthermore, the T-2 toxin has been reported to induce oxidative stress and inflammation, thereby causing organ toxicity. Studies have confirmed that T-2 toxin can increase the excessive production of nitric oxide by increasing various inflammatory cytokines, thus leading to mitochondrial damage and redox imbalance, further inducing apoptosis (Liu et al., 2017). Although T-2 toxin is an environmental risk factor for KBD, the potential mechanism by which T-2 toxin damages articular cartilage remains unknown. In the current study, a series of experiments was carried out to clarify the potential mechanism of damaged cartilage induced by T-2 toxin.

Our results demonstrated T-2 toxin-induced pathological changes in the articular cartilage of rats. The KEGG pathway enrichment results showed that IL-17 and TNF signaling pathways were related to inflammation. In addition, T-2 toxin-related genes obtained from GeneCards were used for enrichment analysis, and it was found that the IL-17 signaling pathway was also significantly expressed. These results indicated that the inflammatory reaction may play an important role in the damaged joint cartilage of rats induced by T-2 toxin. In addition, Mmp3, Tnf, Mapk10, Ccl11, Creb5, Cxcl2, and Cebpb were significantly enriched in the IL-17 and TNF signaling pathways.

Interleukin-17 (IL-17) is a member of a new family of inflammatory cytokines (Amatya et al., 2017). IL-17 is associated with many inflammatory diseases, such as rheumatoid arthritis, asthma, lupus, and allograft rejection (Aggarwal & Gurney, 2002; Moseley et al., 2003; Kolls & Lindén, 2004). IL-17 receptors are widely distributed in various tissues and participate in the activation of transcription factor NF-κB and the kinase JNK pathway, which are related to inflammatory and immune diseases (Schwandner et al., 2000; Moseley et al., 2003). Tumor necrosis factor (TNF) is mainly produced by activated macrophages and T lymphocytes, and proto-TNF is expressed on the plasma membrane, where it can be cleaved by matrix metalloproteinases (MMPs) in the extracellular region, thus releasing its soluble form (Bradley, 2008). It is well known that pro-inflammatory cytokines including IL-1b, TNF, and IL-6 promote cartilage degradation by stimulating the production of MMPs (Zhou et al., 2014). A proteinase activation cascade is likely initiated by plasmin, the product of plasminogen activator activity, which in turn activates latent stromelysin (MMP3), an activator of latent collagenases (Goldring, 2000). Activation of the NLRP3 inflammasome leads to a wide range of immune responses, including the production of pro-inflammatory cytokines and chemokines and cell death (Wree et al., 2013; Broderick et al., 2015). Studies have shown the liver inflammation and fibrosis induced by the NLRP3 inflammasome were affected by IL-17 and TNF, and the effect of TNF was more significant (Wree et al., 2018). Therefore, some inflammasomes in the articular cartilage may also be affected by IL-17 and TNF, which leads to the enhancement of the inflammatory response. MAPK10 is a member of the Jun N-terminal kinase subgroup in the mitogen-activated protein kinase superfamily and is involved in multiple signaling pathways in key physiological processes, such as apoptosis, differentiation, and proliferation (Davis, 2000; Yan et al., 2018). Some studies have shown that MAPK10 plays a central role in atrial fibrillation in mice, and MAPK10 knockout can significantly reduce atrial inflammation (Liu et al., 2022). The chemokine C-C motif ligand 11 (CCL11) is a member of the CC chemokine family and is produced by connective tissue cells and leukocytes in the human body (Wakabayashi et al., 2021). CCL11 can induce the migration of many types of leukocytes, including eosinophils, basophils, macrophages, and dendritic cells (Elsner et al., 1996; Peled et al., 1998; Menzies-Gow et al., 2002). The chemokine CXCL2 is induced by macrophage toll-like receptor signal transduction and is responsible for the recruitment of neutrophils, which is the most important step in response to pathogens (De Filippo et al., 2008). Our transcriptome sequencing results showed that the expression of Cxcl2 was downregulated. Therefore, it can be speculated that T-2 toxin may reduce the ability of rats to resist pathogens by inhibiting the expression of Cxcl2.

In addition, the IHC analysis results showed that the expression levels of Mmp3 and Tnf proteins in the T-2 toxin group were significantly higher than those in the control group. This indicated that T-2 toxin can activate the expression of Mmp3 and Tnf, which is consistent with the RNA-seq results. Generally, TNF first binds to its receptors and then transmits molecular signals for biological functions such as inflammation and cell death. It has also been shown that T-2 toxin can increase the level of TNF (Lin et al., 2019). TNF can activate the immune system, but inappropriate or excessive production of TNF may be harmful and lead to rheumatoid arthritis, inflammatory bowel disease, psoriasis arthritis, psoriasis, and non-infectious uveitis (Jang et al., 2021). TNF inhibitors have been successfully developed and applied in the clinical treatment of autoimmune diseases such as Crohn’s disease and rheumatoid arthritis (Raychaudhuri & Raychaudhuri, 2009; Lis et al., 2014). MMP3 is a protease that is synthesized and secreted by synovial fibroblasts and chondrocytes, and it can be used as a reliable marker of rheumatoid arthritis (Lerner et al., 2018). The MMP3 enzyme can degrade a variety of collagen, proteoglycans, fibronectin, laminin, and elastin. In addition, it can activate other MMPs, such as MMP-1, MMP-7, and MMP-9. Therefore, MMP3 plays a key role in connective tissue remodeling (Lerner et al., 2018).

In this study, RNA-seq was used to explore the mechanism of damaged cartilage induced by T-2 toxin, which may enhance the inflammatory response by activating the IL-17 and TNF signaling pathways, resulting in an abnormal inflammatory response in chondrocytes and cartilage injury. However, several limitations need to be addressed. First, an animal model was used to study the inflammatory response involved in damaged articular cartilage, and this needs to be validated in the cartilage of KBD patients. Second, further molecular experiments should be performed to validate the IL-17 and TNF signaling pathways. Despite these limitations, our results provided new insight for further understanding the mechanism of damaged articular cartilage induced by T-2 toxin.

## Data Availability

The original contributions presented in the study are included in the article/Supplementary Material; further inquiries can be directed to the corresponding author.
